# Supplementary material for: DMRforPairs: identifying Differentially Methylated Regions between unique samples using array based methylation profiles
Source: BMC Bioinformatics. 2014 May 15;15:141. doi: 10.1186/1471-2105-15-141 (PMC4046028; doi:10.1186/1471-2105-15-141)
Supplement: Additional file 2 — DMRforPairs output for the comparison of A431-MCF7 and NA17018-NA17105. Please start from the HTML files in each folder. Available via the BMC Bioinformatics website. [file 1471-2105-15-141-S2.zip › 1394847754114233_MOESM2_ESM/A431_MCF7/figures/28556.pdf]

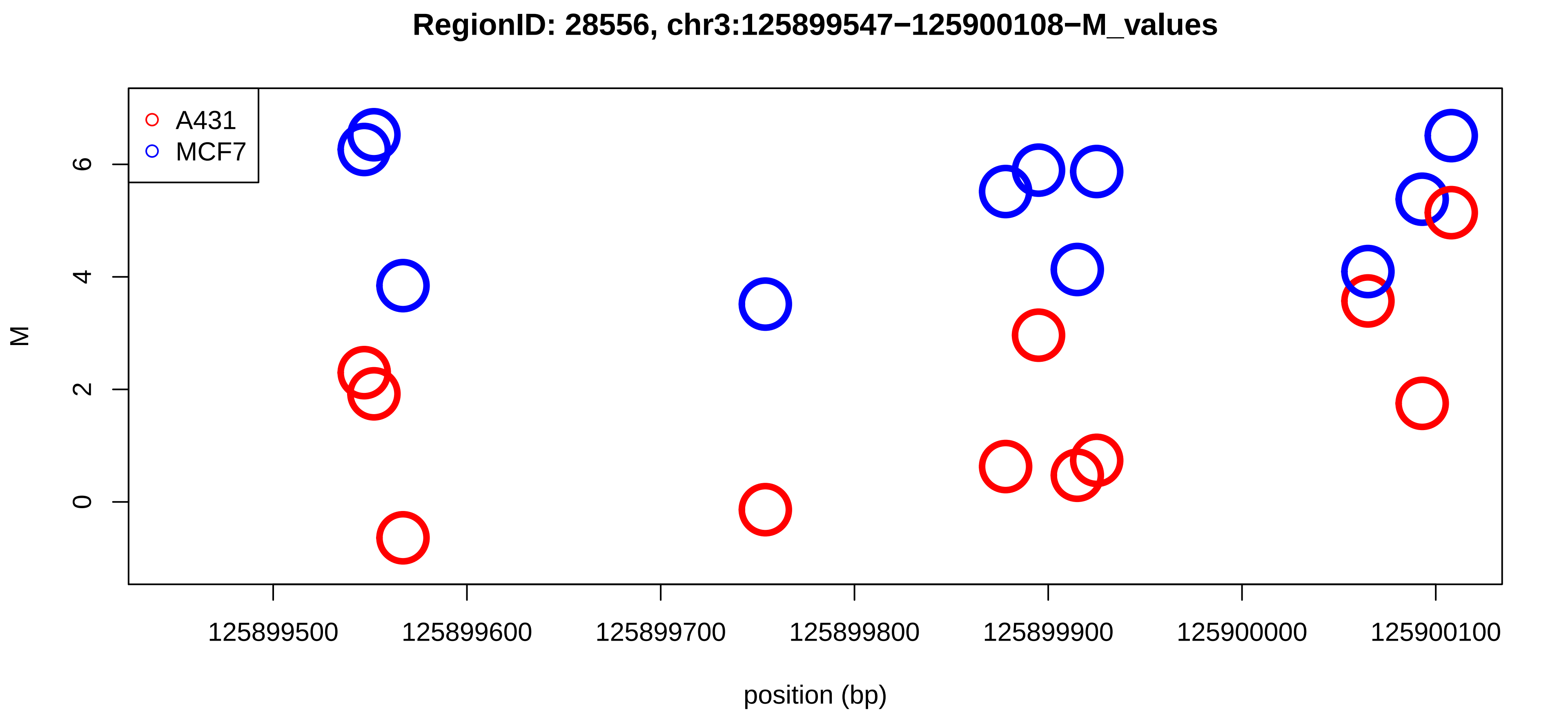

RegionID: 28556, chr3:125899547−125900108−M_values
A431
MCF7
M
position (bp)
125899500
125899600
125899700
125899800
125899900
125900000
125900100

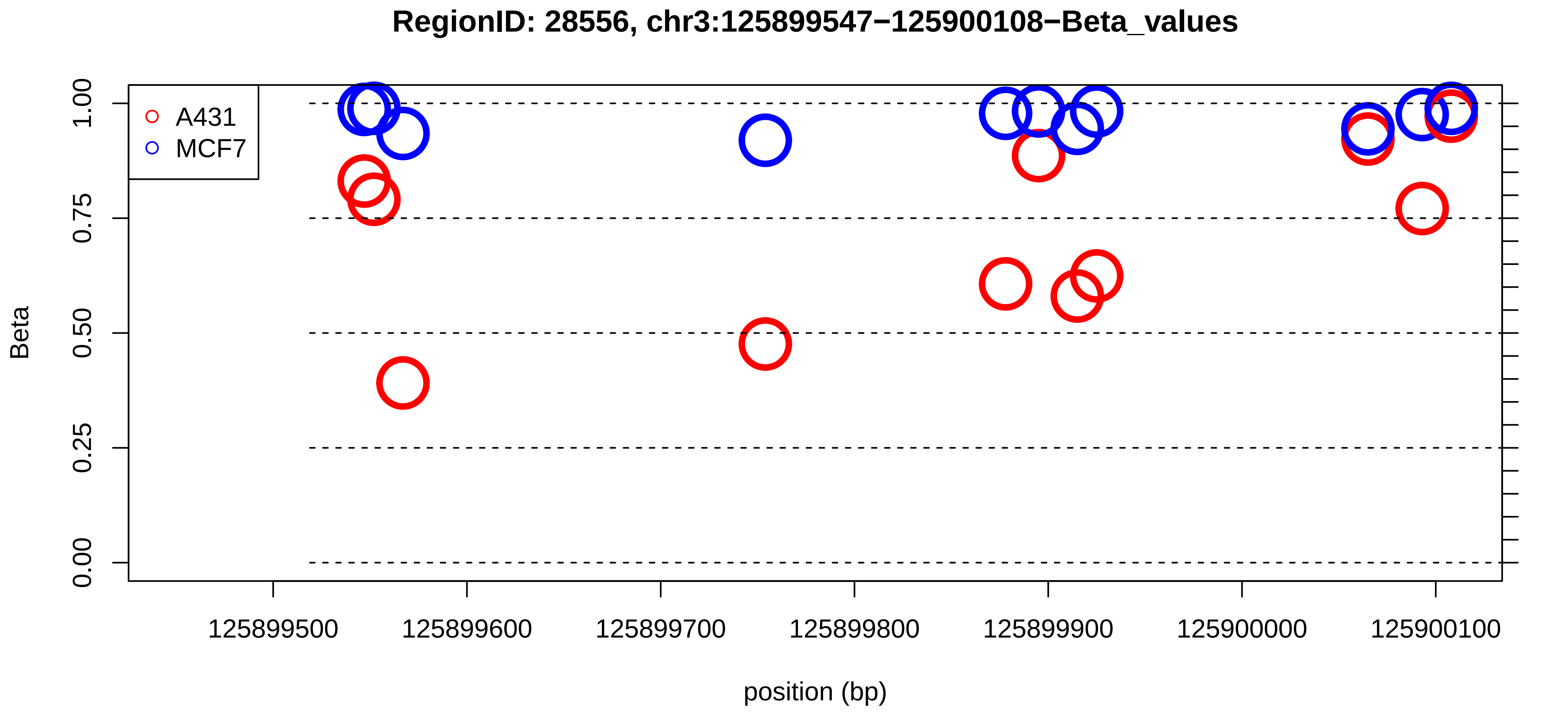

RegionID: 28556, chr3:125899547−125900108−Beta_values
A431
MCF7
Beta
position (bp)
125899500
125899600
125899700
125899800
125899900
125900000
125900100

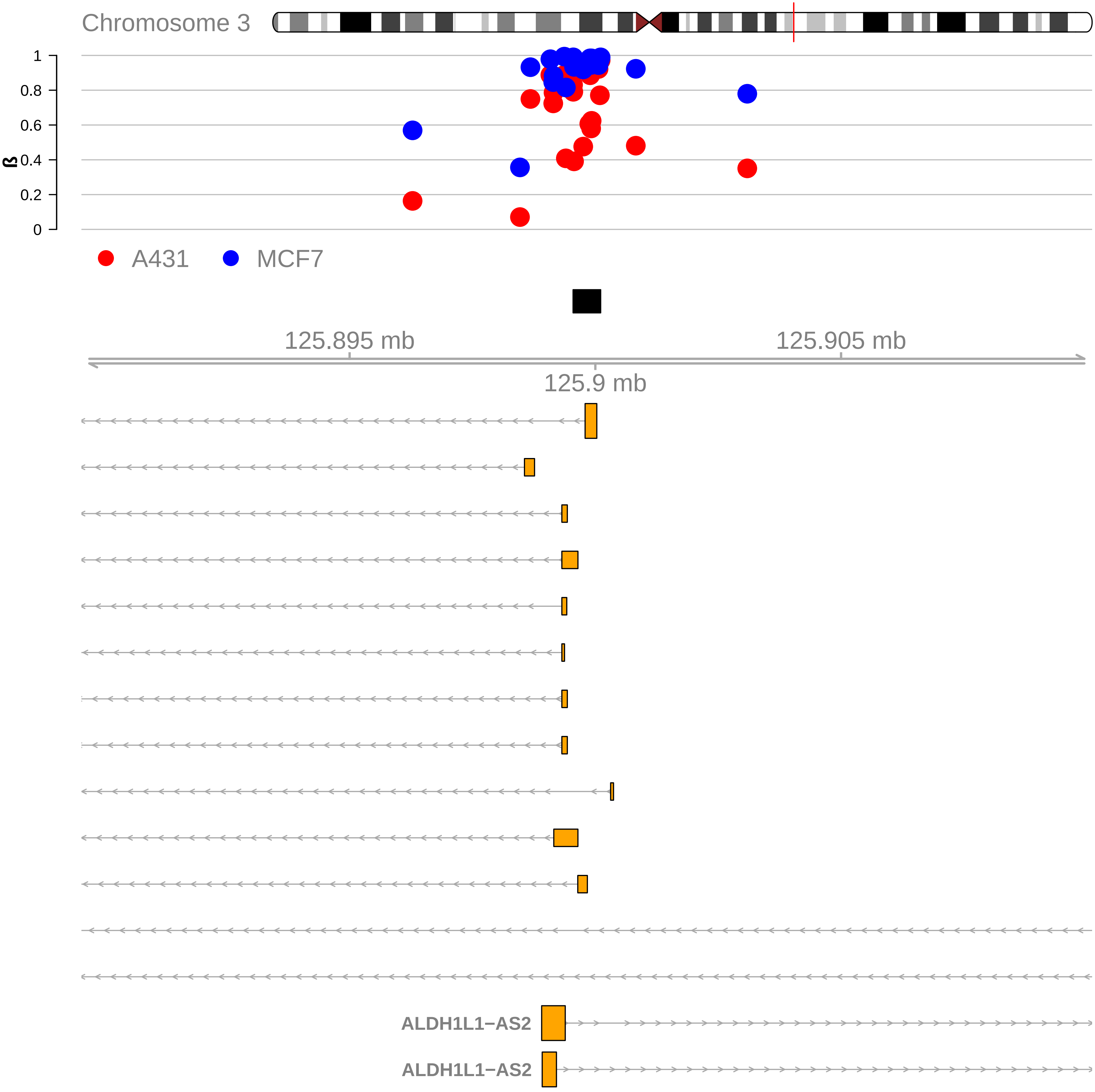

Chromosome 3
1
0.8
0.6
ß
0.4
0.2
0
A431
MCF7
125.895 mb
125.905 mb
125.9 mb
ALDH1L1−AS2
ALDH1L1−AS2
